# The Complement Anaphylatoxin C3a Receptor (C3aR) Contributes to the Inflammatory Response in Dextran Sulfate Sodium (DSS)-Induced Colitis in Mice

**DOI:** 10.1371/journal.pone.0062257

**Published:** 2013-04-26

**Authors:** Elisabeth Wende, Robert Laudeley, André Bleich, Eva Bleich, Rick A. Wetsel, Silke Glage, Andreas Klos

**Affiliations:** 1 Institute for Medical Microbiology and Hospital Epidemiology, Hannover Medical School, Hannover, Germany; 2 Institute for Laboratory Animal Science, Hannover Medical School, Hannover, Germany; 3 Brown Foundation Institute of Molecular Medicine, University of Texas, Health Science Center, Houston, Texas, United States of America; Charité-University Medicine Berlin, Germany

## Abstract

Inflammatory bowel diseases are a critical public health issue, and as treatment options remain limited, there is a need to unravel the underlying pathomechanisms in order to identify new therapeutic targets. Complement activation was found in patients suffering from inflammatory bowel disease, and the complement anaphylatoxin C5a and its receptor C5aR have been implicated in disease pathogenesis in animal models of bowel inflammation. To further characterize complement-related pathomechanisms in inflammatory bowel disease, we have investigated the role of the anaphylatoxin C3a receptor in acute dextran sulfate sodium-induced colitis in mice. For this, colitis was induced in C3a receptor-deficient BALB/c and C57BL/6 mice, and disease severity was evaluated by clinical and histological examination, and by measuring the mRNA expression or protein levels of inflammatory mediators in the tissue. C3a receptor deficiency was partially protective in BALB/c mice, which had significantly reduced weight loss, clinical and histological scores, colon shortening, and CXCL-1/KC mRNA, myeloperoxidase and interleukin-6 tissue levels compared to the corresponding wild type mice. In C57BL/6 mice the differences between wild type and C3a receptor-deficient animals were much smaller and reached no significance. Our data demonstrate that the contribution of C3a receptor to disease pathogenesis and severity of dextran sulfate sodium-induced colitis in mice depends on the genetic background. Further studies will be required to clarify whether targeting of C3a receptor, possibly in combination with C5a receptor, might be considered as a therapeutic strategy for inflammatory bowel disease.

## Introduction

Inflammatory bowel diseases (IBD), which in humans comprise Crohn’s disease and ulcerative colitis, are chronic recurring inflammatory disorders of the gastrointestinal tract. As they impose a serious burden of suffering on affected individuals and frequently lead to inability to work, IBD are a critical public health issue. The incidence of IBD is increasing, but treatment options remain limited. Much effort has been made to identify the pathomechanisms underlying IBD, and it is believed that inflammation is caused by dysregulated mucosal immune responses to luminal antigens, triggered in susceptible individuals by as yet unidentified environmental factors. Histopathologically, IBD is characterized by mucosal inflammation with crypt distortion and abscesses, ulceration, edema, and recruitment of neutrophils and lymphocytes. The infiltrating immune cells release chemokines and cytokines, which amplify the inflammatory reaction and play a key role in IBD pathogenesis. This issue has been addressed by the development of tumor necrosis factor (TNF)-α-blocking antibodies, which are used in IBD that does not respond to conventional treatment. However, anti-TNF-α therapy is not beneficial for all patients, and may cause immunological side effects [1]. Efforts to block other cytokines have yielded some promising results but their efficacy remains to be validated (reviewed in [2]). To improve the overall treatment options for IBD, the identification of novel therapeutic targets is needed, and in this context, pro-inflammatory components of the complement system might be considered.

Complement is an integral part of the immune system and is activated by contact with foreign surface structures like lipopolysaccharide, mannose, necrotic cells, or by immune complexes. Upon complement activation, a proteolytic cascade is initiated and results in the release of components that contribute to host defense and inflammation. These include the anaphylatoxic peptides C3a and C5a, which mediate pro-inflammatory and immunemodulatory signals via their corresponding seven-transmembrane domain receptors, C3a receptor (C3aR) and C5a receptor (C5aR). The anaphylatoxin receptors are found on myeloid cells like granulocytes, mast cells, dendritic cells, monocytes and macrophages, and on non-myeloid cells such as neurons. In inflammation, they induce cytokine production, degranulation and chemotaxis of leukocytes, and vascular permeability (reviewed in [3]; [4]).

Although the primary functions of the anaphylatoxins are in host defense, their excessive or sustained generation causes tissue damage and adversely affects the course of inflammatory conditions. C3aR is a well-established player in airway hyperresponsiveness, where it aggravates inflammation by promoting a T helper cell (Th)2-polarized response [5]; [6]. Moreover, C3aR has been found harmful in *P. aeruginosa* pneumonia, whereas its functions in sepsis may vary depending on the tissue. C5aR has been shown to play deleterious roles in sepsis, airway hyperresponsiveness, rheumatoid arthritis, neurodegenerative diseases, and ischemia-reperfusion injury (reviewed in [3]). Animal experiments and observations in patients provide evidence for complement activation in IBD, and for its participation in pathogenesis with mainly adverse effects [7]–[10]. Mice deficient in the complement regulatory protein decay-accelerating factor, which protects host cells against assembly of C3 convertases early in the complement cascade, exhibit aggravated dextran sulfate sodium (DSS)-induced colitis [11]. This indicates that downstream complement effectors are involved in bowel inflammation. To this effect, our group has previously investigated the role of C5a/C5aR in a mouse model of IBD, dextran sulfate sodium (DSS)-induced colitis, and found that targeted deletion of C5aR is protective in acute but disadvantageous in chronic colitis [12]. Others have found C5aR antagonists or C5a antibodies to ameliorate trinitrobenzenesulfonic acid (TNBS)-induced colitis in rats or mice [13]–[15].

Here, we focused on the role of C3aR in IBD, and gave special attention to the influence of the genetic background, by examining C3aR-deficient BALB/c as well as C57BL/6 mice in DSS-induced colitis. We found that C3aR deficiency was partially protective in BALB/c mice but not significantly protective in C57BL/6 mice, showing that the effect of C3aR on bowel inflammation depends on the genetic background.

## Materials and Methods

### Animals and Housing

Wild type BALB/c (BALB/c WT) and C57BL/6 (B6 WT) mice were obtained from Janvier SAS (Le Genest-Saint-Isle, France) and Charles River Laboratories (Wilmington, MA, USA), respectively. Breeding pairs of C3aR-deficient mice (C.129S4-C3ar1^tm1^Cge/J; BALB/c *C3ar^-/-^*) were purchased from The Jackson Laboratory (Bar Harbor, ME, USA). Breeding pairs of C3aR-deficient C57BL/6 mice (B6.129X1-C3ar1^tm1^Raw, B6 *C3ar^-/-^*) were provided by Prof. Dr. R. A. Wetsel (Institute of Molecular Medicine, The University of Texas, Houston, TX, USA). Mice were housed and bred under specific pathogen-free conditions. Routine microbiological monitoring was carried out according to FELASA recommendations [16]. This study was conducted in accordance with the German Animal Protection Law and with the European Communities Council Directive 86/609/EEC for the protection of animals used for experimental purposes. All experiments were approved by the Local Institutional Animal Care and Research Advisory Committee and permitted by the local authority (Lower Saxony State Office for Consumer Protection, Food Safety, and Animal Welfare Service, AZ/permit number: 33.9-42502-04-09/1705).

### DSS-induced Colitis

Male mice aged 8–10 weeks were used. Starting four weeks before colitis induction, litter was exchanged weekly between all involved cages to distribute the specific pathogens present in the facility to all mouse strains and to avoid acute infections as confounding variables during the experiment. Group sizes for each independent experiment were between 4–10 per mouse line, limited by the number of offspring available at any one time. In parallel to B6 *C3ar^−/−^* other C57BL/6 lines without relevance to this manuscript were tested, and B6 WT mice were always included as controls. For this reason, the overall number of mice tested was larger for B6 WT than for the other mouse lines. To induce intestinal inflammation, mice were fed 3% DSS, M_W_ 36–50 kDa (MP Biomedicals, Illkirch, France) *ad libitum* with the drinking water from day 0 through day 7. Body weight and clinical signs of disease were recorded beginning on day -1. To account for inaccuracies in weight determination, a mean initial weight was calculated from the first three measurements on days -1, 0, and 1, during which the values were stable, and the body weight for days 2–7 was determined relative to the mean initial weight (in %) for each individual. The following criteria contributed to the clinical score (score points in brackets): coat (0–3); posture (0–3), spontaneous behavior (0–3), induced behavior (0–3), feces (0–2), hygiene of the perianal region (0–2), overall health appearance (0–3). According to the research permit, a DSS concentration was chosen that should not lead to clinical scores ≥15. If on rare occasions individual mice scored ≥15, they were euthanized before the end of the experiment to minimize suffering. On day 7, mice were sacrificed, blood was withdrawn by cardiac puncture, colon length was determined, and mesenteric lymph nodes (mLN), cecum, and colon tissue samples were collected for further analysis.

### Determination of Colon Length

Mean colon length differed between C57BL/6 and BALB/c (p<0.001 for all groups with t-test): 10.6 cm or 0.46 cm/kg body weight for BALB/c WT, 8.0 cm or 0.34 cm/kg body weight for B6 WT mice. To make the degree of DSS-induced colon shortening directly comparable between the two strains, colon length was expressed as a percentage. For this, colon length, measured from the distal end of the caecum to the anus, was put in relation to the initial weight for each mouse, to account for length variations that might be due to individual body size. To compare DSS-treated WT and *C3ar^−/−^* groups, the mean colon length from the respective H2O-treated control groups was set to 100%. For each of the DSS-treated mice, the colon length was then calculated as percentage of the control group mean, and the means and SD were determined of those percentage values.

### Histology

The cecum and colon were fixed in 4% formaldehyde, embedded in paraffin, cut into 4–6 µm sections, and stained with hematoxylin and eosin. Histological scoring was done in a blinded fashion and followed a scheme adapted from Cooper *et al.* [17] and Bleich *et al.* [18], including the following criteria: epithelial changes (crypts unchanged: 0, loss of basal ⅓: 1, basal ⅔: 2, complete: 3, loss of crypts plus ulceration: 4), cellular infiltration (none: 0, infiltrates in lamina propria: 1, in lamina propria plus edema formation: 2, in lamina propria and submucosa: 3), area involved (none: 0, 10–30%: 1, 40–60%: 2, >60%: 3; applied to each of the parameters). This scheme yielded scores from 0–13 for each segment (cecum; proximal, medial, and distal colon), i.e. 0–39 for total colon and 0–52 for colon plus cecum. Images of histological sections were taken on an Axioskop 40 microscope (Carl Zeiss AG, Oberkochen, Germany).

### Quantitative Real Time PCR

For RNA extraction, the RNAII kit (Macherey Nagel, Duren, Germany) was used. Colon tissue samples were preserved in RNAlater™ (Qiagen, Hilden, Germany) and homogenized with ¼” ceramic spheres in Fastprep® tubes (MP Biomedicals, Illkirch, France) in the supplied buffer on a Ribolyzer instrument (Hybaid, Middlesex, UK). For reverse transcription with Superscript™ II (Invitrogen, Darmstadt, Germany), 5 µg of total RNA and 250 ng random hexamer oligonucleotides (Roche, Mannheim, Germany) were used. Gene expression was analysed with the qPCR Core kit for SYBR®-Green (Eurogentec, Köln, Germany) using the following primers: forward 5′-CTGGTGAAAAGGACCTCTCG-3′, reverse 5′-GTCAAGGGCATATCCAACAAC-3′ for Hprt-1 (*Hprt1,* NM013556.1); forward 5′-GCACCCAAACCGAAGTCATA-3′, reverse 5′-TGGGGACACCTTTTAGCATC-3′ for CXCL-1/KC (*Cxcl1*, NM008176); forward 5′-GGGTATGTCTCTCTGTGTAG-3′, reverse 5′-GGAGAAGATTAGGGGAGAAC-3′ for CCL-8 (*Ccl8*, NM021443.1); forward 5′-CAAATCCCTCAAAGACCTCAA-3′, reverse 5′-CTTTTGCTTTTTCTTTTGGCTG-3′ for CXCL-9 (*Cxcl9*, NM008599.2); and forward 5′-GAGATGCTCACTTTGACGATG-30, reverse 5′-CCTTGGCTGAGTGGTAGAG-3′ for matrix metalloproteinase 3 (*Mmp3*, NM010809.1). A serial dilution of pooled cDNA was used as a standard, and expression of all genes was normalized to expression of the housekeeping gene *Hprt1*. Gene induction in DSS-treated mice was calculated as -fold change in expression compared to the control group.

### Quantification of Inflammatory Mediators

Colon tissue samples were snap-frozen in liquid nitrogen and homogenized as described above, in 10 µL/mg tissue of sample buffer (200 mM NaCl, 4 mM EDTA, 10 mM Tris, 10% [w/v] glycine, cOmplete Protease Inhibitor Cocktail (Roche), pH 7.4). To determine myeloperoxidase (MPO), an enzyme-linked immunosorbent assay (ELISA) kit for mouse myeloperoxidase (HyCult Biotechnology, Uden, Netherlands) was used. Cytokine levels were measured with a Mouse Th1/Th2/Th17 Cytokine Cytometric Bead Array™ (CBA) Kit (BD, Heidelberg, Germany) on a FACSCalibur™ flow cytometer (BD), and data was analysed using FCAP Array software (BD).

### C3a ELISA

For the detection of C3a/C3a-desArg by ELISA, purified rat anti-mouse C3a antibody (clone I87–1162, BD), and biotinylated rat anti-mouse C3a (clone I87–419, BD) antibody were employed as described elsewhere [12]. As a standard sample, zymosan-activated EGTA plasma was included in each run. Additionally, purified murine C3a (BD Pharmingen, Heidelberg, Germany) was used as a standard to calculate actual peptide concentrations. Of note, neoepitope-specific antibodies directed against C3 cleavage products (here: ELISA capture antibody) are usually much less sensitive for C3a than for C3a-desArg (as discussed in [3]). In contrast to the standard of C3a, the zymosan-activated standard plasma as well as the plasma samples mainly contain C3a-desArg. Thus, the calculated and depicted concentrations for C3a are most likely (10–100 fold?) false high. Specificity of the C3a/C3a-desArg ELISA was confirmed using zymosan-activated EGTA plasma from C3-deficient mice as a negative control.

### Statistical Analysis

For statistical analyses, SigmaStat 3.5 (Systat Software, Chicago, IL, USA) was used. Clinical and histological scores were evaluated by Mann-Whitney *U*-test. Differences in parametric data were analysed by Student’s *t*-test where applicable, otherwise by Mann-Whitney *U*-test. Differences of P<0.05 were considered significant.

## Results

### Partially Protective Effect of C3aR Deficiency in BALB/c Mice in Acute DSS-induced Colitis

**Figure 1:**
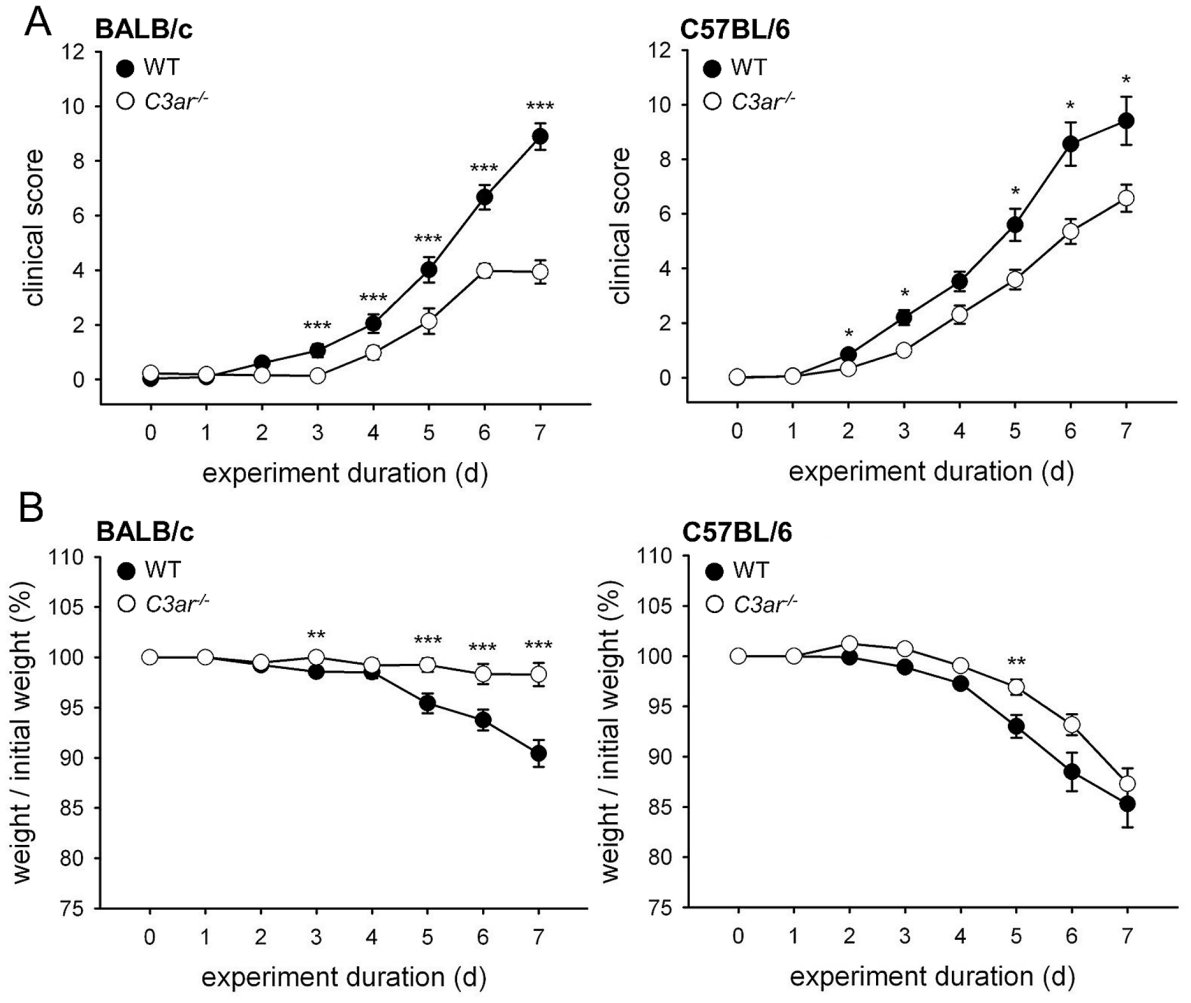
Clinical course of DSS-induced colitis in *C3ar^-/-^ vs.* WT BALB/c and B6 mice. Mice were given 3% DSS with the drinking water from day 0–7. Clinical score and weight were determined daily, starting on day –1. Control animals fed with water did not have any clinical signs or weight loss (data not shown). **A**, Time course of clinical score and **B**, Time course of body weight. To minimize suffering, three out of 25 B6 WT animals had to be sacrificed on day 6, leading to a seemingly smaller increase of the clinical score, and to a seemingly smaller weight loss, from day 6 to day 7, as compared to the preceding days. The data shown are means ± SEM of two independent trials with a total of n = 33 WT mice and n = 32 *C3ar^-/-^* mice for BALB/c, and of four independent trials with a total of n = 25 (day 0–6) or n = 22 (day 7) WT mice and n = 21 *C3ar^-/-^* mice for C57BL/6. *, p<0.05; **, p<0.01; ***, p<0.001.

To gain insight into the role of C3aR in bowel inflammation on different genetic backgrounds, BALB/c and B6 mice deficient in C3aR were subjected to DSS-induced colitis. Clinical signs of illness appeared from day 2 on (Fig. 1A), starting with traces of blood in the feces, next followed by diarrhoea, coat aberrances, bent posture, and later also by reduced spontaneous agility, and weak resistance to provocation. In BALB/c *C3ar^-/-^* mice, the appearance of first disease signs was delayed until day 4, and in both B6 *C3ar^-/-^* and BALB/c *C3ar^-/-^* mice, clinical scores from day 2 to day 7 were lower than in the corresponding WT animals. From day 3–4 on, clinical signs were accompanied by weight loss, reaching ∼15% in B6 WT, and ∼10% in BALB/c WT on day 7 (Fig. 1B). In contrast, the body weight of BALB/c *C3ar^-/-^* mice remained nearly stable, and was significantly higher than in BALB/c WT mice on days 5–7. Between B6 *C3ar^-/-^* and B6 WT animals, no significant differences in body weight were found. Due to a clinical score >15 and/or weight loss >30%, three out of 25 B6 WT animals had to be sacrificed on day 6, so they were not available for scoring and weighing on day 7, and their tissues were excluded from analysis. In the graphical representation, this appears as a seemingly smaller increase of the clinical score from day 6 to day 7, as compared to the preceding days (Fig. 1A), and a seemingly smaller weight loss, respectively (Fig. 1B). Consequently, the determinable differences between B6 WT and the less affected B6 *C3ar^-/-^* mice were smaller in these and probably also in other parameters. Taken together, these data indicated that in DSS-induced colitis, C3aR deficiency is moderately protective, which was more apparent in BALB/c than in B6 mice.

### Reduced Complement Activation in BALB/c C3ar^-/-^ Mice in DSS-induced Colitis

**Figure 2:**
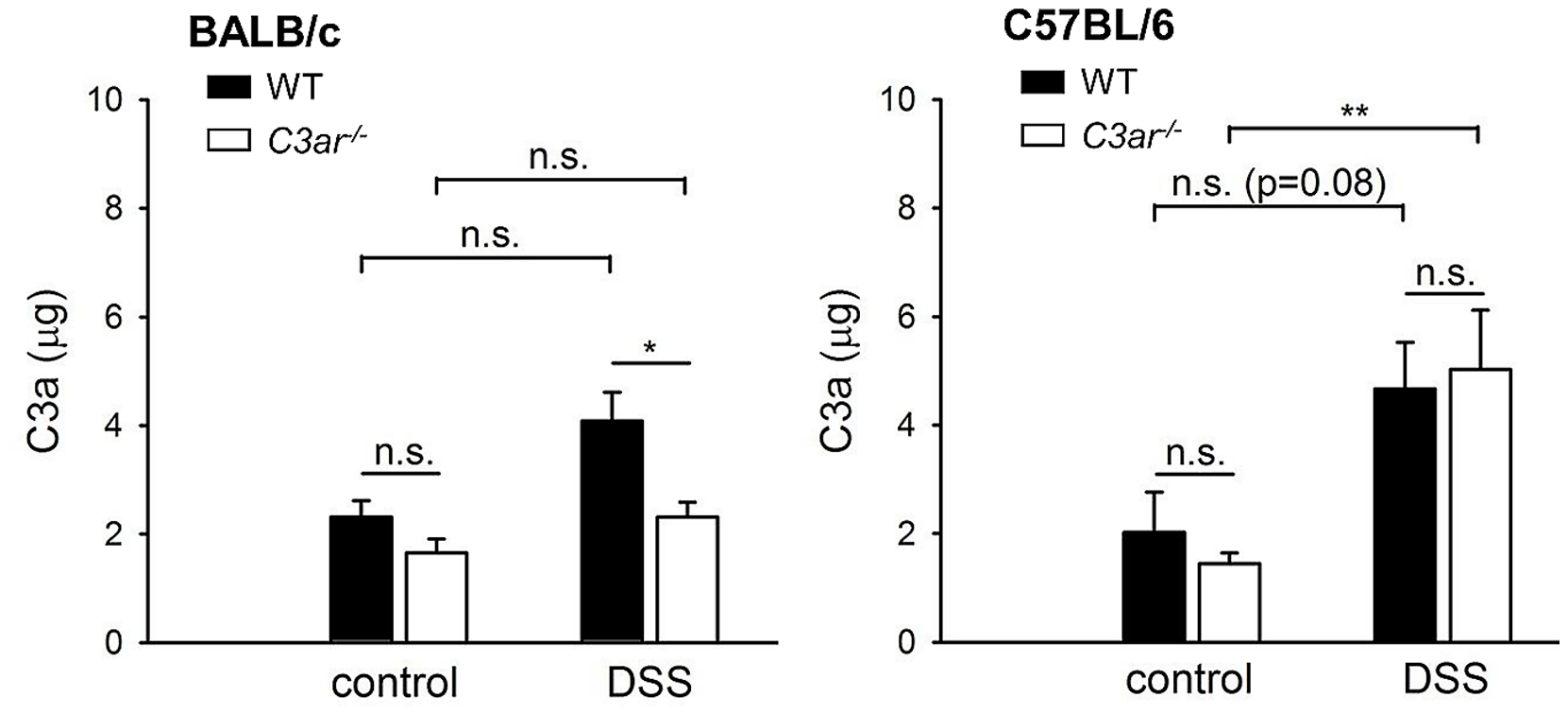
Plasma C3a/C3a-desArg levels in *C3ar^-/-^ vs.* WT BALB/c and B6 mice in DSS colitis. C3a/C3a-desArg was measured by enzyme-linked immunosorbent assay (ELISA) in EDTA plasma samples withdrawn by cardiac puncture. Of note, the calculated and depicted values are most likely too high because purified murine C3a had to be used as standard (see Materials and Methods). The data shown are means ± SEM from n = 4 (WT, H_2_O), n = 21 (WT, DSS), n = 4 (*C3ar^-/-^*, H_2_O), and n = 21 (*C3ar^-/-^*, DSS) mice for BALB/c, and n = 4 (WT, H_2_O), n = 11 (WT, DSS), n = 4 (*C3ar^-/-^*, H_2_O) and n = 12 (*C3ar^-/-^*, DSS) mice for C57BL/6. n.s., not significant; *, p<0.05.

To assess complement activation, C3a and its degradation product C3a-desArg were measured in plasma samples taken on day 7. After DSS-induced colitis, C3a plasma levels were moderately elevated in B6 *C3ar^-/-^* mice only (Fig. 2). This excludes high systemic activation and suggests that complement activation was mainly restricted to the inflamed bowel. Measurement of C3a/C3a-desArg in colon tissue homogenate returned equally elevated high levels for all groups including H_2_O controls and thus did not provide additional information, probably attributable to complement activation during the preparation of this tissue.

### C3aR Deficiency Slightly Reduces Macroscopic Pathology, Histological Alterations, and Tissue Myeloperoxidase Content in BALB/c Mice

**Figure 3:**
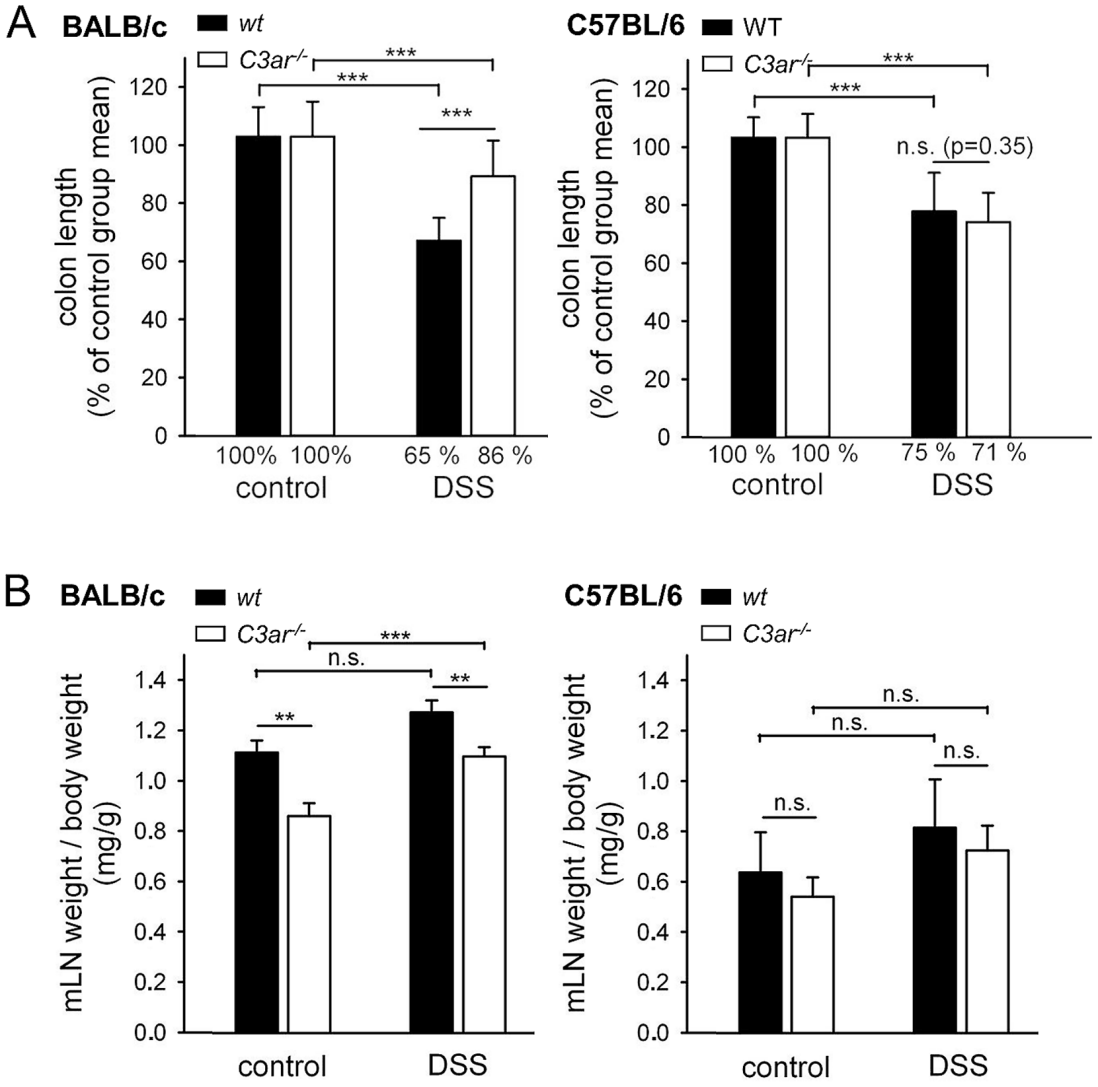
Macroscopic findings in *C3ar^-/-^ vs.* WT BALB/c and B6 mice in DSS colitis. To account for individual variations in body size, colon length and mLN weight were calculated relative to the body weight for each mouse. **A**, Colon shortening. To compare DSS-treated WT and *C3ar^-/-^* mice, the mean colon length from the H_2_O-treated control groups was set to 100%, and used as a relative measure. **B**, Mesenteric lymph node (mLN) weight. The data shown are means ± SD (colon shortening) or ± SEM (mLN weight) of two independent trials with a total of n = 16 (WT, H_2_O), n = 33 (WT, DSS), n = 16 (*C3ar^-/-^*, H_2_O), and n = 32 (*C3ar^-/-^*, DSS) mice for BALB/c, and of four independent trials with a total of n = 15 (WT, H_2_O), n = 25 (WT, DSS, day 0–6) or n = 22 (WT, DSS, day 7), n = 12 (*C3ar^-/-^*, H_2_O), and n = 21 (*C3ar^-/-^*, DSS) mice for C57BL/6. n.s., not significant; ***, p<0.001.

In DSS-induced colitis, colon shortening is regarded as a macroscopic measure of disease pathology and tissue remodelling in inflammation [19]; [20]. As shown in Fig. 3A, DSS treatment led to shortening of the colon in all four mouse lines. In BALB/c WT mice, this was significantly more pronounced than in BALB/c *C3ar^-/-^* mice, whereas no difference was seen between B6 WT and B6 *C3ar^-/-^*. Along with the colon, the mesenteric lymph nodes (mLN) were removed, and their weight was determined. BALB/c *C3ar^-/-^* mice had smaller mLN than their WT counterparts, both without and with DSS treatment. In B6 mice there were only non-significant differences in mLN size between WT and *C3ar^-/-^*. After DSS colitis mLN size was significantly increased in BALB/c *C3ar^-/-^* mice and non-significantly increased in the other three lines (Fig. 3B).

**Figure 4:**
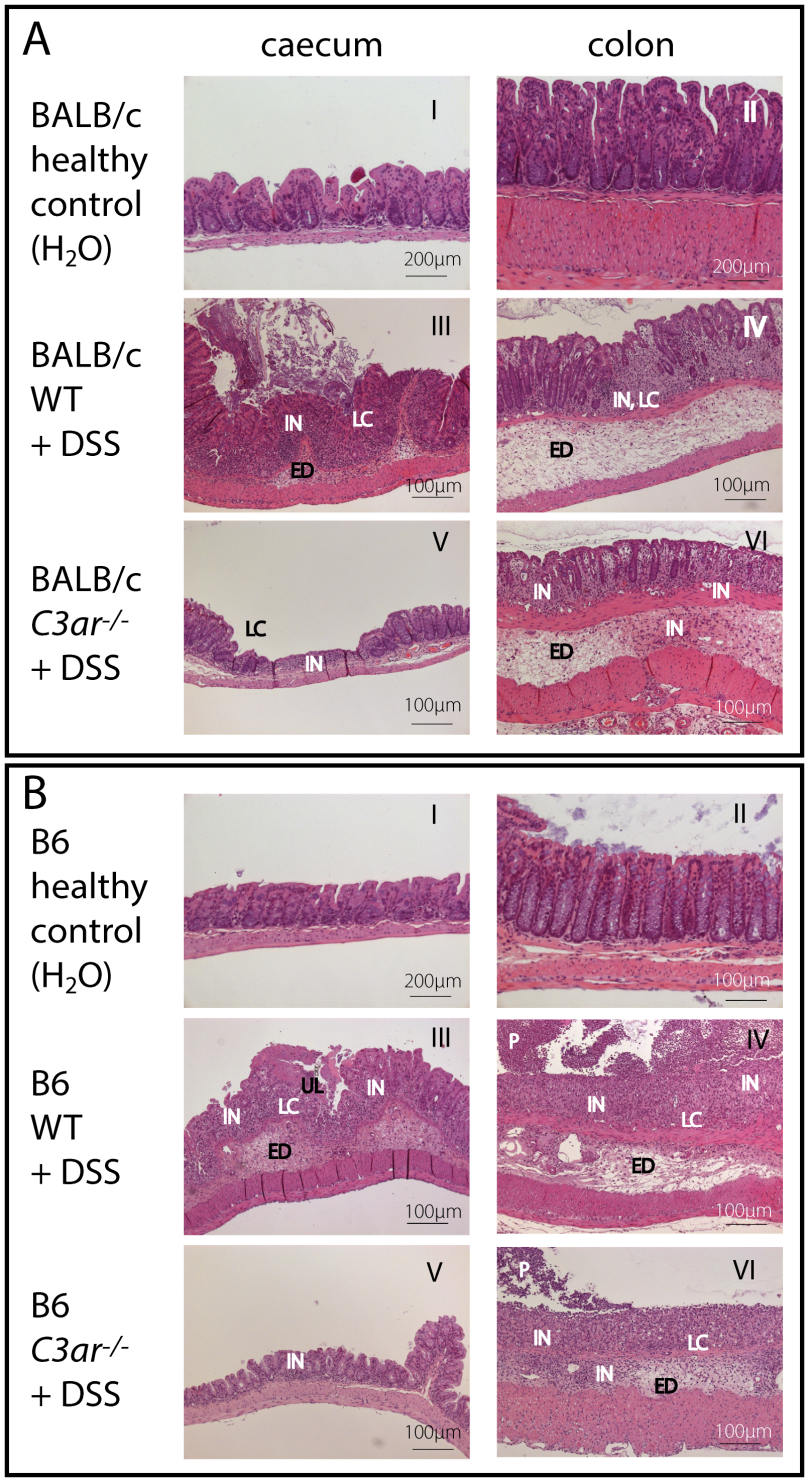
Histological evaluation of *C3ar^-/-^ vs.* WT BALB/c and B6 mice in DSS colitis (first part). **A** (BALB/c) and **B** (C57BL/6), Representative sections of the cecum and colon stained with hematoxylin and eosin. Enlargement of the keratinized epithelium was found exclusively and epithelial hyperplasia was more frequent in BALB/c WT mice. ED = edema, HY = epithelial hyperplasia, IN = inflammatory cell infiltrate, KE = enlargement of keratinized epithelium, LC = loss of crypts, P = pus, UL = ulceration. See also Fig. **5 C** for histological score based on the severity and extent of inflammatory changes in the cecum and colon. No histological abnormalities were found in control animals fed with water (data not shown).

**Figure 5:**
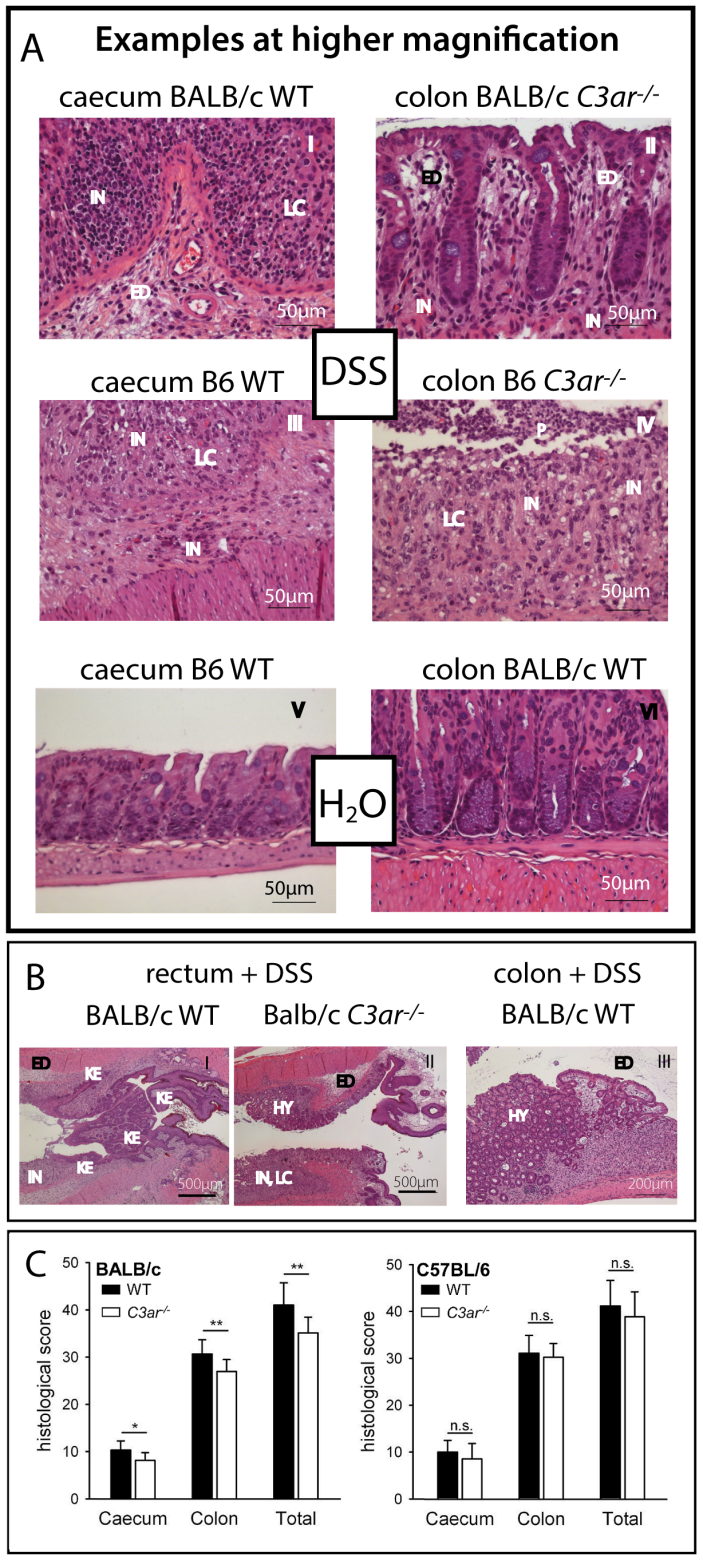
Histological evaluation of *C3ar^-/-^ vs.* WT BALB/c and B6 mice in DSS colitis (second part). Sections of the cecum, colon and rectum stained with hematoxylin and eosin, and histological score, as indicated. **A**, Exemplary lesions (edema, cellular infiltrates, loss of crypts) found in DSS-treated animals. Note that the details are not representative of the overall severity or the frequency of the lesions in the respective treatment group. **B**, Exemplary sections of the rectum of DSS-treated BALB/c WT and *C3ar^-/-^* mice. Enlargement of the keratinized epithelium was found exclusively and epithelial hyperplasia was more frequent in BALB/c WT mice. ED = edema, HY = epithelial hyperplasia, IN = inflammatory cell infiltrate, KE = enlargement of keratinized epithelium, LC = loss of crypts, P = pus, UL = ulceration. **C**, Histological score (as depicted in Fig. 4 and 5A and 5B) based on the severity and extent of inflammatory changes in the cecum and colon. For each specimen, the colon score is the sum of the scores for the proximal, medial and distal parts, and the total score is the combined score of the cecum and colon. The data shown are means ± SD of specimens from n = 11 (WT, DSS) and n = 10 (*C3ar^-/-^*, DSS) mice for BALB/c, and n = 24 (WT, DSS) and n = 10 (*C3ar^-/-^*, DSS) mice for C57BL/6. No histological abnormalities were found in control animals fed with water (data not shown). n.s., not significant; *, p<0.05; **, p<0.01.

Histological examination of the cecum and colon revealed inflammatory tissue damage in all DSS-treated animals (Fig. 4 and 5). The epithelium was affected by loss of crypt architecture, crypt abscesses, and ulcerations (Fig. 4A and 4B, panels III-VI, and Fig. 5A). In the lamina propria and in the submucosal tissue, edema formation and infiltrates of mononuclear (*i.e.* lymphocytes and macrophages) and polymorphonuclear cells (*i.e.* granulocytes) were frequent (Fig. 5A). In all mouse lines, epithelial alterations, cellular infiltrates and ulcerations were most pronounced in the medial colon, followed by the distal colon and cecum, and finally by fewer and less extensive lesions in the proximal colon. Especially in BALB/c WT mice, areas of mucosal hyperplasia and intramucosal edema in the colon were observed (Fig. 5B, panel III), together with a dense infiltrate of inflammatory cells even into the submucosal muscular layer (not shown). Notably, the rectum sections of six out of the 11 examined BALB/c WT animals displayed enlargement of the keratinized epithelium, which was neither found in BALB/c *C3ar^-/-^* mice (Fig. 5B, panels I and II), nor in C57BL/6 mice (not shown). Overall, BALB/c *C3ar^-/-^* mice were slightly but significantly less affected than BALB/c WT mice (Fig. 5C), whereas between B6 WT and *C3ar^-/-^* mice, no differences were found.

**Figure 6:**
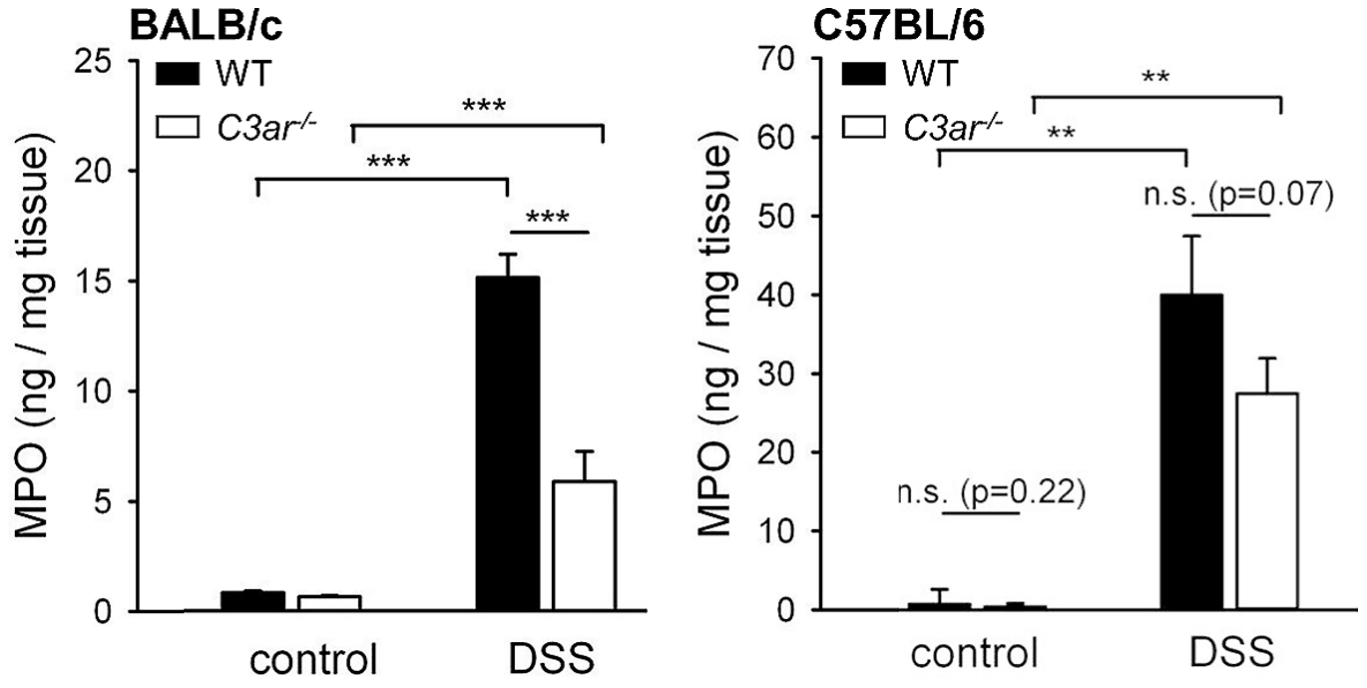
Myeloperoxidase (MPO) levels in the colonic tissue of *C3ar^-/-^ vs.* WT BALB/c and B6 mice in DSS colitis. Levels of the granulocyte marker enzyme MPO were measured by enzyme-linked immunosorbent assay (ELISA) in tissue homogenates. The data shown are means ± SEM from n = 7 (WT, H_2_O), n = 21 (WT, DSS), n = 5 (*C3ar^-/-^*, H_2_O), and n = 21 (*C3ar^-/-^*, DSS) mice for BALB/c, and n = 5 (WT, H_2_O), n = 11 (WT, DSS), n = 5 (*C3ar^-/-^*, H_2_O) and n = 12 (*C3ar^-/-^*, DSS) mice for C57BL/6. n.s., not significant; ***, p<0.001.

To quantify the degree of inflammatory cell infiltration, the levels of myeloperoxidase, an enzyme produced mainly by neutrophil granulocytes, were measured in the colon tissue. After DSS treatment, BALB/c *C3ar^-/-^* mice had significantly lower tissue MPO concentrations than BALB/c WT mice (Fig. 6), and B6 *C3ar^-/-^* had slightly lower MPO levels than B6 WT mice, but without significance. Remarkably, DSS-treated B6 WT and *C3ar^-/-^* mice had about 2.5– and 4.5–fold higher colon tissue MPO levels than the respective BALB/c groups. Flow cytometric analysis of whole blood samples from healthy WT mice showed that this was not due to strain differences in circulating granulocyte levels: BALB/c WT even had slightly higher numbers of circulating granulocytes than B6 WT mice (data not shown).

Overall, C3aR deficiency significantly reduced disease pathology in BALB/c mice, while in B6 mice, no significant effects of C3aR were observed.

### Differential Cytokine Profile in the Colon Tissue of BALB/c and B6 Mice, and Differential Effects of C3aR Deficiency

To further characterize the inflammatory response, colon tissue homogenates were analysed for protein and transcript levels of inflammatory mediators that may be regulated during DSS-induced colitis [12].

**Figure 7:**
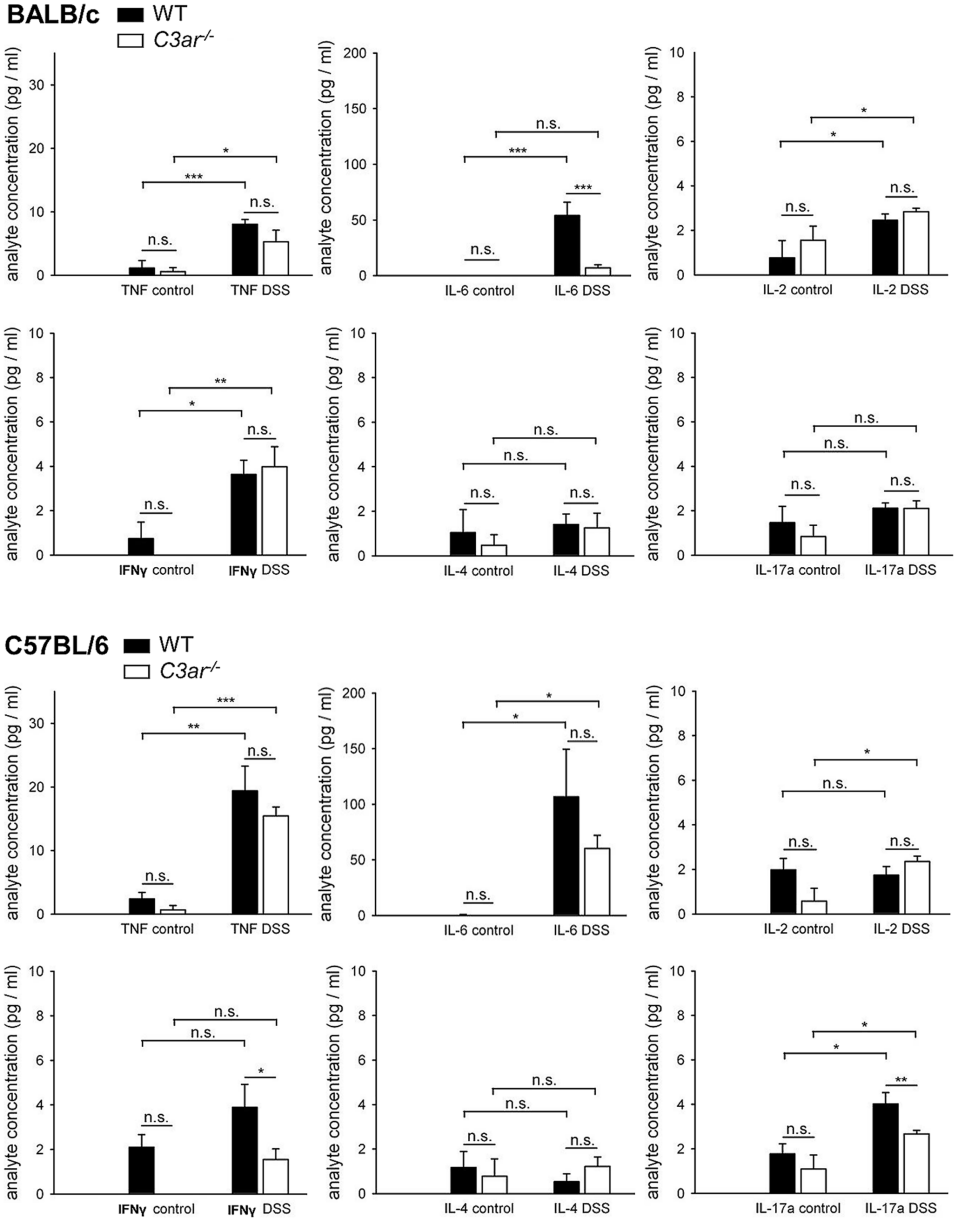
Cytokine profile of *C3ar^-/-^ vs.* WT BALB/c and B6 mice in DSS colitis. Cytokine levels were measured in tissue homogenates using a bead-based immunoassay. The data shown are means ± SEM from n = 3 (WT, H_2_O), n = 10 (WT, DSS), n = 5 (*C3ar^-/-^*, H_2_O), and n = 11 (*C3ar^-/-^*, DSS) mice for BALB/c, and n = 6 (WT, H_2_O), n = 10 (WT, DSS), n = 4 (*C3ar^-/-^*, H_2_O) and n = 11 (*C3ar^-/-^*, DSS) mice for C57BL/6. n.s., not significant; *, p<0.05; **, p<0.01; ***, p<0.001.

On the protein level, the pro-inflammatory cytokines TNF-α and interleukin (IL)-6 were elevated in DSS-treated BALB/c WT, B6 WT, and B6 *C3ar^-/-^* mice (Fig. 7). In BALB/c *C3ar^-/-^* mice, TNF-α levels but not IL-6 levels were elevated, leading to significantly lower levels of IL-6 in DSS-treated BALB/c *C3ar^-/-^ vs*. WT mice. In B6 *C3ar^-/-^*, IL-6 levels were non-significantly lower than in B6 WT, and in both BALB/c *C3ar^-/-^* and B6 *C3ar^-/-^*, TNF-α levels were slightly yet non-significantly lower than in the respective WT mice. The cytokine IL-2, which induces T cell proliferation, was slightly increased in all DSS-treated groups except B6 WT, but there were no differences between WT and *C3ar^-/-^* animals. To assess the induction of Th1, Th2, and Th17 cells during colitis, the levels of the corresponding cytokines interferon (IFN)-γ, IL-4, and IL-17A were determined. After DSS treatment, IFN-γ was comparably increased in BALB/c WT and *C3ar^-/-^* mice, but not significantly increased in B6 mice (Fig. 7). DSS-treated B6 *C3ar^-/-^* had slightly lower IFN-γ levels than B6 WT mice. IL-4 levels were not elevated in any of the DSS-treated groups. IL-17A was not elevated in BALB/c mice, but in B6 mice, and B6 *C3ar^-/-^* had lower IL-17A levels than B6 WT mice after DSS-induced colitis. The levels of the immunomodulatory cytokine IL-10 were below the detection limit in all groups (data not shown).

**Figure 8:**
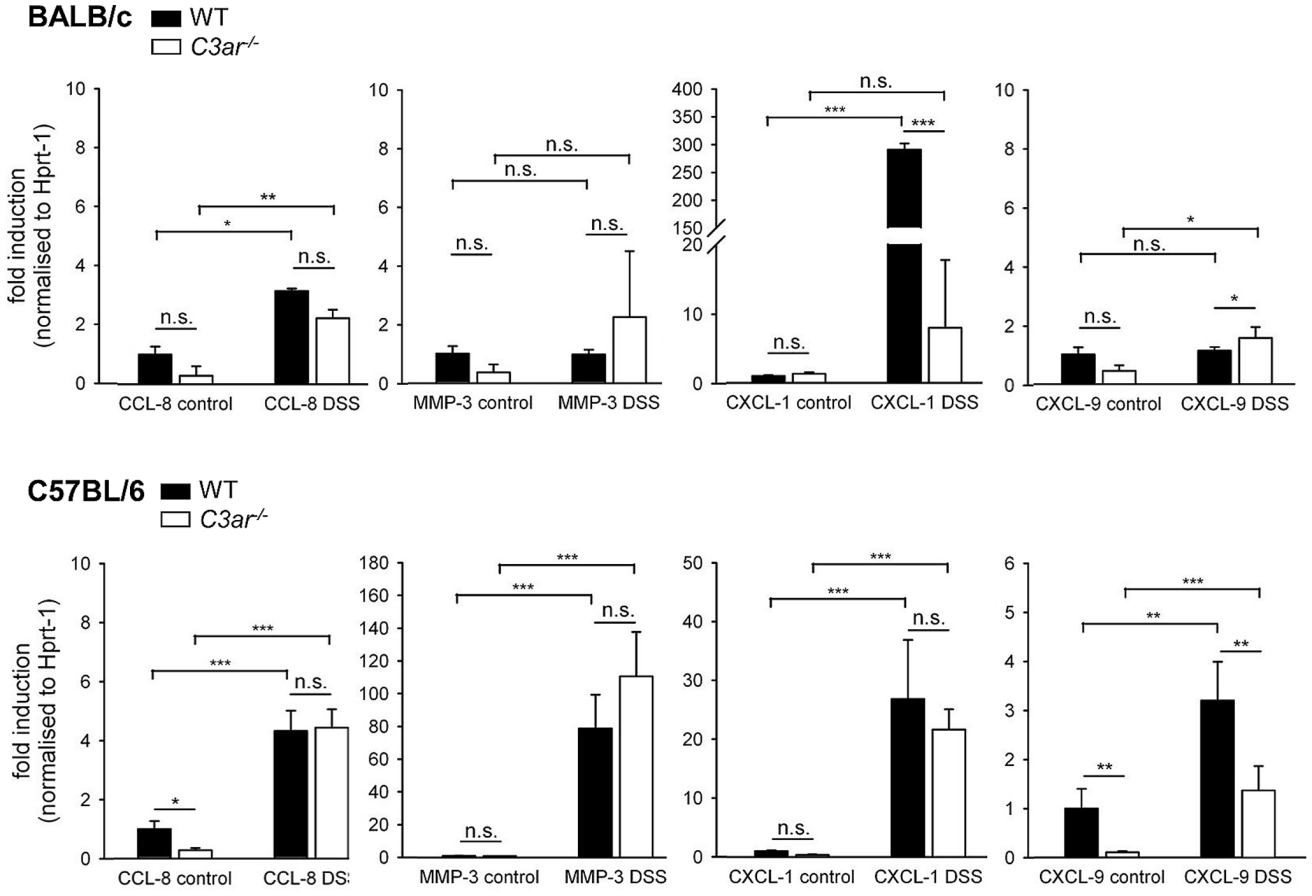
Regulation of mRNA expression in *C3ar^-/-^ vs.* WT BALB/c and B6 mice in DSS colitis. RNA was isolated from colon tissue homogenates, and mRNA levels were measured by quantitative real time PCR and normalized to the expression of the housekeeping gene *Hprt-1*. For each gene, mRNA expression is shown relative to its expression in the WT control group. The data shown are means ± SEM from n = 12 (WT, H_2_O), n = 22 (WT, DSS), n = 12 (*C3ar^-/-^*, H_2_O), and n = 21 (*C3ar^-/-^*, DSS) mice for BALB/c, and n = 12 (WT, H_2_O), n = 10 (WT, DSS), n = 12 (*C3ar^-/-^*, H_2_O), and n = 14 (*C3ar^-/-^*, DSS) mice for C57BL/6. *, p<0.05; **, p<0.01; ***, p<0.001.

TNF-α and IFN-γ are known to induce the expression of other pro-inflammatory mediators, including CXCL-1/KC, CCL-8, CXCL-9 and MMP-3, through transcriptional regulation. In DSS-treated BALB/c WT and *C3ar^-/-^* mice, quantitative real time PCR analysis revealed slight upregulation of mRNA for CCL-8, a chemoattractant for various types of immune cells that is implicated in IBD [21], and unchanged levels of mRNA for MMP-3, a protease in tissue remodelling, including during bowel inflammation [22]; [23] (Fig. 8). There were significant differences in the regulation of mRNA for the neutrophil chemotaxin CXCL-1/KC, which was substantially elevated in BALB/c WT but not in BALB/c *C3ar^-/-^* mice after DSS administration. Minor upregulation of T cell chemoattractant CXCL-9 mRNA [24] was found in DSS-treated BALB/c *C3ar^-/-^* but not in WT mice. In both DSS-treated B6 WT and *C3ar^-/-^* mice, there were moderate increases in CCL-8 mRNA, and in CXCL-1/KC mRNA (Fig. 8). Unlike BALB/c mice, both B6 wt and *C3ar^-/-^* mice showed substantial upregulation of MMP-3 mRNA after DSS-induced colitis. CXCL-9 mRNA was upregulated in colitis in B6 mice, but the effect of C3aR on the expression of this cytokine was difficult to interpret, as B6 WT and *C3ar^-/-^* mice had different basal levels of CXCL-9 mRNA.

Thus, BALB/c and B6 mice develop partially different cytokine profiles during DSS-induced colitis, and C3aR appears to differentially contribute to their regulation.

## Discussion

Crohn’s disease and ulcerative colitis, the two major forms of IBD in humans, have a severe impact on health and quality of life of the affected individuals. In many cases, the currently available treatments cannot ameliorate the disease for lasting periods of time, or cause serious side effects. To establish novel therapeutic strategies in the long run, a broader knowledge of the pathomechanisms underlying intestinal inflammation is needed. These are now widely accepted to include a barrier dysfunction of the intestinal epithelium, and an overdriven immune response to luminal antigens.

Among the diverse rodent models of bowel inflammation, none may reflect all aspects of human IBD pathogenesis, but still many of them have proven useful to elucidate involved pathways and have contributed to the development of novel therapeutics. In several murine models of colitis, T cells were identified as the driving force of inflammation, and those models are highly suitable to investigate therapeutics that target T cell function [25].

In contrast, DSS-induced colitis is initiated by epithelial damage and independently of T or B cells [26]. However, innate immune mechanisms and cells that contribute to human IBD are activated early in DSS-induced colitis, and T cells are involved later in the inflammation process [12]; [27]–[29]. The DSS-induced colitis model has contributed to identify candidate drugs and drug targets, including TNF-α, which is now an established therapeutic target in human IBD [30]; [31]. In our group, the DSS-induced colitis model was previously employed to demonstrate an adverse role for C5aR, and the resulting data were in good accordance with those that other investigators obtained from TNBS-induced colitis, and from tissue samples of IBD patients [12]; [15]. In acute DSS-induced colitis in mice, the complement anaphylatoxin receptor C5aR has detrimental functions, and C3aR, C5aR, and the alternative receptor for C5a, C5L2, are upregulated in the colon tissue [12].

Here, we investigated the role of C3aR in experimental IBD, and the influence of the genetic background, by comparing BALB/c and B6 WT and *C3ar^-/-^* mice in acute DSS-induced colitis. We found that the complement system was activated in both mouse strains during DSS-induced colitis. After DSS treatment, BALB/c *C3ar^-/-^* had significantly lower plasma C3a levels than BALB/c WT mice. This may be attributable to an attenuation of a positive feedback loop of local inflammation and tissue damage, partially driven by the anaphylatoxins and their receptors, on complement activation. On the C57BL/6 background, where markers of inflammation were less influenced by the gene knockout, plasma C3a levels did not differ between DSS-treated WT and *C3ar^-/-^* mice.

Clinical and histological scores were similar in B6 WT and BALB/c WT mice, while complement activation and tissue levels of the pro-inflammatory cytokines TNF-α and IL-6 were slightly higher in B6 WT than in BALB/c WT mice. In the colonic tissue of DSS-treated BALB/c WT mice, we found an increase in IFN-γ, and in B6 WT there was non-significant upregulation of IFN-γ as well as significant upregulation of IL-17A. This is in accordance with other studies that have reported a Th1-polarized immune response in BALB/c mice [26]; [32], and a mixed Th1/Th17 profile for C57BL/6 mice [29] in acute DSS-induced colitis.

In BALB/c mice, the absence of C3aR was protective, as evidenced by a delayed onset of clinical signs, less weight loss, and reduced histological damage in BALB/c *C3ar^-/-^* mice after colitis. In BALB/c *C3ar^-/-^*, the tissue levels of the granulocyte marker enzyme MPO, and of the inflammatory cytokine IL-6, were lower than those in BALB/c WT animals. However, in B6 mice treated with DSS, there were no obvious differences between *wt* and *C3ar^-/-^* animals. B6 *C3ar^-/-^* had improved clinical scores over B6 WT mice, but regarding weight loss, histology, and MPO and IL-6 levels, there was no amelioration.

Notably, neutrophil influx in terms of MPO levels in the colonic tissue was higher in B6 WT than in BALB/c WT mice, despite BALB/c WT having slightly higher levels of circulating granulocytes. However, mRNA levels of the neutrophil chemoattractant CXCL-1/KC were much more abundant in BALB/c WT mice. This might indicate that in BALB/c mice, CXCL-1/KC is less efficient in neutrophil recruitment, or that in C57BL/6 mice, other compensating chemoattractants make greater contributions. Neutrophils are critically involved in tissue damage in DSS-induced colitis, as shown by a study in which the exacerbated phenotype of macrophage- and/or dendritic cell-depleted mice in DSS-induced colitis was rescued by antibody-mediated neutrophil removal [28]. However, in CXCL-1/KC-deficient mice, where tissue infiltration is dominated by mononuclear cells, the inflammatory response in DSS-induced colitis is increased [33], suggesting that a certain amount of neutrophil recruitment is required for adequate inflammatory and regenerative responses in this disease model. We found significantly reduced levels of CXCL-1/KC mRNA and of MPO in BALB/c *C3ar^-/-^ vs.* WT mice after DSS administration, and a non-significant reduction of MPO in B6 *C3ar^-/-^ vs.* WT mice. C3a/C3aR thus appears to contribute to CXCL-1/KC mRNA production in acute DSS-induced colitis, and to augment neutrophil infiltration. Neutrophil recruitment might be more dependent on CXCL-1/KC in BALB/c mice than in B6 mice, which could explain why the impact of C3aR deficiency becomes apparent on the BALB/c background only.

Levels of CCL-8 mRNA were upregulated in all DSS-treated groups, suggesting that this chemotaxin for various immune cells contributes to DSS-induced bowel inflammation but is not influenced by C3a/C3aR. MMP-3 mRNA levels were not affected by DSS in BALB/c mice, whereas in DSS-treated C57BL/6 mice they were substantially elevated without significant difference of WT *vs*. *C3ar^-/-^*. This may correlate with the higher levels of neutrophils and inflammatory cytokines in C57BL/6, reflecting a strain-specific inflammatory reaction. In BALB/c, other metalloproteases that were not examined here may participate in tissue remodelling.

Apart from direct inflammatory effector cells such as granulocytes and phagocytes, Th cell subsets play an important role in orchestrating the immune response in IBD models (reviewed in [25]; [34]). We observed that BALB/c *C3ar^-/-^* mice had smaller mLN than BALB/c WT independent of DSS treatment, which might be due to lower numbers of lymphocytes residing in the mLN. Others reported that B6 *C3ar^−/−^* mice had fewer and less viable T cells in their spleens than B6 WT mice, and attributed this to the lack of co-stimulatory C3a/C3aR signals for naïve T cells [35]. However, we did not find any significant differences in mLN size between B6 *C3ar^-/-^* and WT mice. After DSS treatment, mLN were significantly enlarged in BALB/c *C3ar^-/-^*, and non-significantly enlarged in B6 *C3ar* ^-/-^, suggesting that substantial expansion of lymphocytes does occur in C3aR-deficient mice. Accordingly, the levels of the T cell cytokine IL-2 were elevated in both BALB/c *C3ar* ^-/-^ and B6 *C3ar* ^-/-^ mice after colitis induction. Another study by Strainic *et al.* reports that C3aR and C5aR signalling is involved in the differentiation of Th17 *vs*. regulatory T cells, and that the functions of the two receptors are overlapping but not redundant [36]. As C5aR plays a prominent role in DSS colitis, it is conceivable that generation of C5a during bowel inflammation is able to partly compensate for the lack of C3a/C3aR signalling, leading to T cell recruitment and activation. Still, signalling by C3a/C3aR might influence the relative abundance of T cell subsets, thus leading to different outcomes in WT *vs*. *C3ar^-/-^* in DSS colitis. An effect of C3aR on the steady state abundance or distribution of immune cells might also underlie the reduced background of some cytokines (mRNA or protein) in *C3ar^-/-^ vs.* WT mice in the absence of disease.

A study that monitored BALB/c and C57BL/6 mice for 4 weeks after 5 days of DSS administration found that BALB/c mice quickly recovered, whereas C57BL/6 mice developed chronic inflammation [37], and in another study, chronification of DSS-induced colitis in C57BL/6 mice was associated with a switch from a Th1/Th17 phenotype to a predominantly Th2-mediated immune response [29]. In our study, DSS-treated B6 *C3ar^-/-^* mice had lower IL-17A and IFN-γ levels than B6 WT, indicating that C3aR participates in the regulation of T cell-mediated responses in DSS-induced colitis. In accordance with their lower IFN-γ levels, B6 *C3ar^-/-^* also had lower mRNA levels than B6 WT mice of the macrophage-derived IFN-γ-inducible T cell chemoattractant CXCL-9. Whether the absence of C3aR might facilitate the Th phenotype switch that initiates the chronic phase of disease in C57BL/6 mice can only be speculated about, as there was no concomitant increase in the Th2 cytokine IL-4, and the lower IL-17A and IFN-γ cytokine levels may simply reflect an overall lower degree of inflammation. However, in BALB/c *C3ar^-/-^* mice, we saw no influence on IL-17A and IFN-γ levels of C3aR, despite its overall more distinctive effect.

In analogy to C5aR, which was shown to modify Th cell polarization in chronic DSS-induced colitis [12], effects of C3aR on cytokine expression may also play a role in chronic disease. For the present study, we initially intended to apply the chronic disease model, but the effects of C3aR found in the acute model are rather small. At present, we therefore consider the chronic model not justifiable in terms of animal welfare.

In conclusion, C3aR contributes to disease pathogenesis in acute DSS-induced colitis in BALB/c mice, although the effect of C5aR may be more prominent [12]. Moreover, the C3aR-induced effects depend on the genetic background, as they are not obvious in C57BL/6 mice. This may be attributable to the different inflammatory profiles that have been reported for the two mouse strains [26]; [29]; [32], and that were also observed by us. Variations in DSS susceptibility between different inbred strains of mice [37]; [38] might also modulate the impact of C3aR on the disease. Given the well-established disadvantageous role for C5aR, and the efficacy of C5aR antagonists and anti-C5a antibodies in acute DSS- and TNBS-induced colitis in rodents [12]–[15], it may be of interest to assess if C3aR and C5aR co-operate in IBD pathogenesis.

Although it is difficult to predict the relevance and therapeutic value of C3aR in human IBD patients with a highly heterogeneous genetic background, it might be worthwhile to assess in the animal model if blockade of C3aR along with C5aR in intestinal inflammation could improve the effect of targeting C5aR alone.
